# Disease‐free survival as a predictor of overall survival in localized renal cell carcinoma following initial nephrectomy: A retrospective analysis of Surveillance, Epidemiology and End Results‐Medicare datac

**DOI:** 10.1111/iju.15104

**Published:** 2023-02-14

**Authors:** Naomi B. Haas, Yan Song, Jaqueline Willemann Rogerio, Su Zhang, Christopher Carley, JingJing Zhu, Rituparna Bhattacharya, James Signorovitch, Murali Sundaram

**Affiliations:** ^1^ University of Pennsylvania Perelman School of Medicine Philadelphia Pennsylvania USA; ^2^ Analysis Group, Inc. Boston Massachusetts USA; ^3^ Merck & Co., Inc. Rahway New Jersey USA

**Keywords:** disease‐free survival, nephrectomy, overall survival, recurrence, renal cell carcinoma

## Abstract

**Objectives:**

This study aimed to assess whether disease‐free survival (DFS) may serve as a predictor for long‐term survival among patients with intermediate‐high risk or high risk renal cell carcinoma (RCC) post‐nephrectomy when overall survival (OS) is unavailable.

**Methods:**

The Surveillance, Epidemiology and End Results‐Medicare database (2007–2016) was used to identify patients with non‐metastatic intermediate‐high risk and high risk RCC post‐nephrectomy. Landmark analysis and Kendall's *τ* were used to evaluate the correlation between DFS and OS. Multivariable regression models were used to quantify the incremental OS post‐nephrectomy associated with increased time to recurrence among patients with recurrence, adjusting for baseline covariates.

**Results:**

A total of 643 patients were analyzed; mean age of 75 years; >95% of patients had intermediate‐high risk RCC at diagnosis; 269 patients had recurrence post‐nephrectomy. For patients with versus without recurrence at the landmark points of 1, 3, and 5 years post‐nephrectomy, the 5‐year OS were 37.0% versus 70.1%, 42.3% versus 72.8%, and 53.2% versus 78.6%, respectively. The Kendall's *τ* between DFS and OS post‐nephrectomy was 0.70 (95% CI: 0.65, 0.74; *p* < 0.001). After adjusting for baseline covariates, patients with one additional year of time to recurrence were associated with 0.73 years longer OS post‐nephrectomy (95% CI: 0.40, 1.05; *p* < 0.001).

**Conclusion:**

The significant positive association of DFS and OS among patients with intermediate‐high risk and high risk RCC post‐nephrectomy from this study supports the use of DFS as a potential predictor of OS for these patients when OS data are immature.

**Abbreviations & Acronyms**
CCI: Charlson Comorbidity Index
DFS: disease‐free survival
DM: distant metastasis
FDA: US Food and Drug Administration
HIPAA: Health Insurance Portability and Accountability Act
HR: hazard ratio
ICD‐O3: International Classification of Disease for Oncology, Third Edition
IPD: individual patient data
LR: locoregional recurrence
NR: not reached
OS: overall survival
RCC: renal cell carcinoma
RMST: restricted mean survival time
SD: standard deviation
SEER: Surveillance, Epidemiology and End Results
TNM: tumor‐node‐metastasis

## INTRODUCTION

Renal cell carcinoma (RCC) is the most prevalent type of kidney cancer, accounting for approximately 90% of cases.^1^ In the United States (US), there were an estimated 73 750 new cases of kidney cancer and 14 830 deaths due to kidney cancer in 2020.^2^ The prognosis for patients with RCC varies based on a range of risk factors such as disease stage, sarcomatoid features, nuclear grade, and necrosis.^3^, ^4^ For patients with newly diagnosed localized, regional, and metastatic disease, the 5‐year overall survival (OS) are 93%, 70%, and 13%, respectively.^1^

Previously, surgical resection (i.e., nephrectomy) was the standard of care for patients with RCC.^5^ However, outcomes were largely dependent on disease stage and grade. The 5‐year OS following nephrectomy of stage 4 RCC was 29.1% with a 10‐year OS of 0%, whereas 5‐ and 10‐year OS in early stage disease was roughly 100%.^6^ Within the last decade, treatment options for advanced RCC have expanded following the introduction of immune checkpoint inhibitors and novel targeted therapies.^7^ The favorable responses associated with the use of these agents have substantially improved the treatment landscape for patients with advanced RCC.^8^

Following the success of targeted therapies in advanced RCC, several clinical trials have been developed to assess their efficacy among primarily resected patients.^9^, ^10^, ^11^, ^12^, ^13^ Common efficacy endpoints used in evaluating adjuvant treatments of RCC have focused on OS and disease‐free survival (DFS). Although OS is the preferred endpoint in oncology, due to the long survival post‐nephrectomy among patients with localized RCC,^14^ mature OS data are not always available for healthcare decision makers at the time of assessment for novel treatments of RCC among primarily resected patients.^15^

Intermediate endpoints, including DFS, have been considered as a predictor for long‐term survival when OS data are immature by regulatory bodies.^16^, ^17^ For example, sunitinib was approved by the US Food and Drug Administration (FDA) in 2017 as an adjuvant therapy for RCC for high risk patients^15^ based on a statistically significant DFS benefit associated with sunitinib versus placebo (6.8 vs. 5.6 years, respectively, *p* = 0.03) from the S‐TRAC trial in the absence of mature OS data.^10^ Evidence based on intermediate endpoints has also been used by payers in making coverage and reimbursement decisions.^18^ A well‐established correlation between intermediate endpoint and long‐term survival based on individual patient data (IPD) can help to further support the use of intermediate endpoints to predict OS by healthcare decision makers, which in turn may accelerate the introduction of innovative treatments for patients.^19^

To the best of our knowledge, no study has investigated the correlation between DFS and OS for patients with RCC post‐nephrectomy using real‐world IPD. To address this knowledge gap, we conducted a retrospective observational study using Surveillance, Epidemiology, and End Results (SEER)‐Medicare linked data to assess the correlation between DFS and OS among patients with intermediate‐high risk and high risk RCC post‐nephrectomy.

## METHODS

### Data source

This retrospective observational study used the SEER‐Medicare database (2007–2016) and included data on patients over the age of 65 years with newly diagnosed, non‐metastatic, intermediate‐high or high risk RCC post‐nephrectomy. The SEER registry data collected demographic, clinical, and mortality data on patients with cancer and the linked Medicare data included Medicare claims for covered health care services from the time of a beneficiary's Medicare eligibility until death. The present study used the linked SEER‐Medicare data available as of November 2018, which include all Medicare‐eligible patients in the SEER data who were diagnosed with cancer through 2015. The data captures Medicare claims through 2016, and Medicare Part D data are available from 2007 onward. All data used in this study were de‐identified and complied with the Health Insurance Portability and Accountability Act (HIPAA) and the Declaration of Helsinki. The New England Independent Review Board exempted this study from institutional board review on January 13, 2020.

### Study population

**FIGURE 1:**
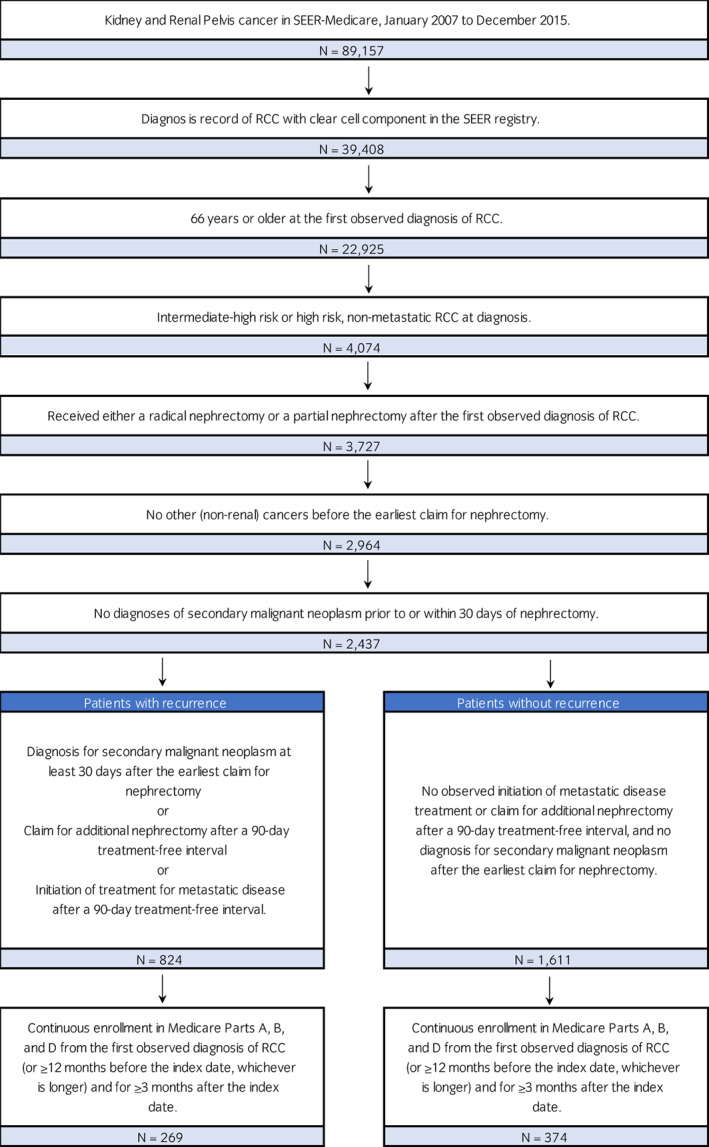
Sample selection. RCC, renal cell carcinoma; SEER, Surveillance, Epidemiology, and End Results.

Patients were eligible for inclusion if they had a diagnosis of RCC between 2007 and 2015 (International Classification of Disease for Oncology, Third Edition [ICD‐O3]: C649 with RCC with clear cell component: ‘8310’). Patients with non‐metastatic RCC were required to be intermediate‐high risk (T2, grade 4, N0, M0 and T3, any grade, N0, M0) or high risk (T4, any grade, N0, M0, and T any stage, any grade, N+, M0) at diagnosis based on the collaborative tumor‐node‐metastasis (TNM) and nuclear grading status. At the time of diagnosis patients were required to be ≥66 years of age, received a partial or a radical nephrectomy after RCC diagnosis, and have no evidence of metastatic disease at diagnosis nor have other non‐renal cancers before nephrectomy. Patients could not be diagnosed with secondary malignant neoplasm prior to, or within 30 days after, the initial nephrectomy. Patients could not be diagnosed with RCC at autopsy, in a nursing home, or by death certificate. Figure 1 summarizes sample selection based on established criteria.

Patients who had recurrence, including locoregional recurrence (LR) and distant metastasis (DM), were further identified. LR was defined as the first diagnosis for secondary disease of intra‐abdominal lymph nodes or kidney and renal pelvis at least 30 days after the earliest claim for nephrectomy or as additional nephrectomy (radical or partial) after the primary treatment‐free period. DM was defined as the first diagnosis for metastatic disease at least 30 days after the earliest claim for nephrectomy or initiation of metastatic RCC treatments following the primary treatment‐free interval. Active treatments for metastatic RCC included commercially available therapies for the treatment of advanced RCC approved by the FDA or recommended from the National Comprehensive Cancer Network. For patients with recurrence, a nephrectomy‐free interval of at least 90 days after the initial nephrectomy was required to indicate the end of primary treatment and serve as a proxy for disease‐free state.^20^, ^21^ Adjuvant therapies were allowed during the nephrectomy‐free interval. For patients with recurrence, the index date was defined as the date 30 days before the indication of recurrence to capture claims that were likely related to but occurred before recurrence.^22^, ^23^ For patients without recurrence, the index date was assigned randomly based on the log‐normal distribution of time between the first nephrectomy and recurrence for patients with recurrence (Figure 1).

Patients were required to be continuously enrolled in Medicare Parts A, B, and D from the first observed diagnosis of RCC, or at least 12 months before the index date, whichever was longer, and for at least 3 months after the index date. The baseline period was defined as 12 months prior to the index date.

### Study outcomes

Study measures included DFS, time to recurrence, and OS. DFS was defined as the time from the initial nephrectomy to recurrence or death, whichever occurred first. OS was defined as the time from initial nephrectomy or other specified landmark point to death. For both DFS and OS, patients were censored at the earliest of loss of follow‐up and end of data availability. Among patients with recurrence, time to recurrence was defined as the time from initial nephrectomy to recurrence.

### Statistical analyses

Continuous baseline variables were described using means and standard deviations (SD); categorical baseline variables were described using frequency counts and percentages. Statistical comparisons of baseline characteristics between patients with and without recurrence were conducted using *t*‐tests for continuous variables and chi‐square tests for categorical variables.

Three different approaches were used to evaluate the association between DFS and OS. Landmark analysis and Kendall's *τ* were used to assess the correlation between DFS and OS post‐nephrectomy. Three landmark points (i.e., 1, 3, and 5 years post‐nephrectomy) were selected to provide the balance of allowing enough time for recurrence to occur while providing enough follow‐up. OS from the corresponding landmark point was described and compared between patients with and without recurrence by these landmark points using KM curves. The hazard ratio (HR) associated with DFS at each landmark point was estimated using multivariable Cox models adjusting for age at nephrectomy, sex, race, disease stage at diagnosis, and Charlson Comorbidity Index (CCI). Kendall's *τ* rank correlation was used to assess the correlation between DFS and OS post‐nephrectomy. Kendall's *τ* was estimated based on a Clayton copula model. A 95% CI and *p*‐value were obtained using a bootstrapping approach with 1000 replications.

Multivariable regression models were used to quantify the incremental OS post‐nephrectomy associated with increased time to recurrence among patients with recurrence based on the pseudovalue regression method.^24^ OS was measured using the restricted mean survival time by the maximum follow‐up time at 10.7‐year post‐nephrectomy. Key patient characteristics, including type of recurrence (i.e., DM vs. LR), year of recurrence, age at nephrectomy, type of nephrectomy (i.e., radical vs. partial), sex, race, CCI, disease stage at diagnosis, and monthly number of all‐cause inpatient and outpatient visits during the baseline period, were adjusted in the main regression. Age at recurrence, instead of age at nephrectomy, was adjusted in a sensitivity analysis to account for the potential indirect effect from time to recurrence to post‐recurrence survival mediated through age.

## RESULTS

### Patient characteristics

A total of 643 patients (269 with recurrence and 374 without) met all inclusion criteria (Figure 1). The mean (±SD) follow‐up duration from the index date was 25.0 (±23.0) months for patients with recurrence and 35.2 (±26.0) months for patients without. The mean age was 75 years, 86% of patients were white and 61% of patients were male (Table 1). Among patients with recurrence, 10.8% had LR and 89.2% had DM. Over 95% of patients had intermediate‐high risk disease at RCC diagnosis. Among patients with intermediate‐high risk RCC at initial diagnosis, over 97% had T3, N0, M0 disease.

**TABLE 1:** Baseline characteristics of patients with RCC recurrence and without recurrence

Baseline characteristics	Patients with recurrence (*N* = 269)	Patients without pecurrence (*N* = 374)	*p*‐Value
Demographic characteristics			
Age (years) at index date, mean ± SD	75.2 ± 6.1	75.7 ± 6.0	0.383
Male, *N* (%)	174 (64.7%)	216 (57.8%)	0.076
White, *N* (%)	231 (85.9%)	323 (86.4%)	0.991
Disease characteristics, *N* (%)			
Year of index date			0.387
2008–2011	86 (32.0%)	116 (31.0%)	
2012–2015	148 (55.0%)	195 (52.1%)	
2016–2017	35 (13.0%)	63 (16.8%)	
TNM staging at diagnosis			0.126
Intermediate‐to‐high risk	>258 (>95.9%)	>363 (>97.0%)	
High risk	<11 (<4.1%)	<11 (<3.0%)	
Recurrence type			—
Locoregional	29 (10.8%)	—	
Metastatic	240 (89.2%)	—	
Comorbidities			
CCI, mean ± SD	3.9 ± 1.7	3.7 ± 1.7	0.242
Comorbidities, *N* (%)			
Hypertension	245 (91.1%)	333 (89.0%)	0.397
Renal disease	136 (50.6%)	176 (47.1%)	0.381
Diabetes	135 (50.2%)	159 (42.5%)	0.054
Chronic pulmonary disease	84 (31.2%)	120 (32.1%)	0.817
Peripheral vascular disease	81 (30.1%)	103 (27.5%)	0.477
Monthly all‐cause HRU, mean ± SD			
Inpatient admissions	0.097 ± 0.098	0.082 ± 0.108	0.068
Emergency department visits	0.064 ± 0.117	0.055 ± 0.115	0.356
Outpatient visits	2.303 ± 1.499	2.052 ± 1.212	0.024
Skilled nursing facility stays	0.014 ± 0.046	0.019 ± 0.118	0.450

*Note*: Statistical comparisons were conducted using *t*‐tests for continuous variables and chi‐square tests for categorical variables.

Abbreviations: CCI, Charlson Comorbidity Index; HRU, healthcare resource utilization; RCC, renal cell carcinoma; SD, standard deviation; TNM, tumor node metastasis.

### Landmark analyses and correlation analysis

**FIGURE 2:**
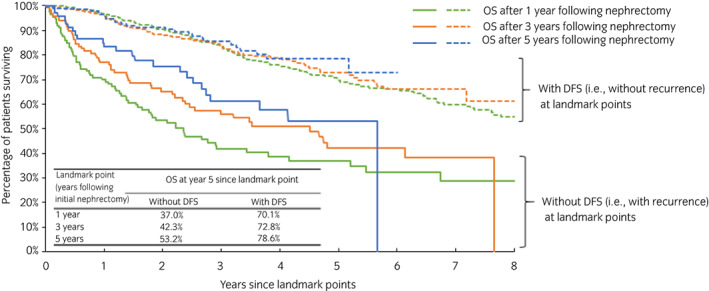
Overall survival stratified by DFS after 1, 3, or 5 years following initial nephrectomy. DFS, disease‐free survival; OS, overall survival.

Patients with recurrence by each landmark point had shorter subsequent OS compared to patients without. For patients with versus without recurrence by 1, 3, and 5 years post‐nephrectomy, the median OS after each landmark point was 2.4 versus 9.7 years, 4.5 versus not reached (NR), and 5.7 versus NR, respectively (all *p* < 0.001) (Table 2). For patients with versus without recurrence, the 5‐year OS 1, 3, and 5 years post‐nephrectomy was 37.0% versus 70.1%, 42.3% versus 72.8%, and 53.2% versus 78.6%, respectively (Figure 2). Patients with recurrence by each landmark point had a significantly increased risk of death following the landmark point (adjusted HR [95% CI]: 1 year post‐nephrectomy: 3.5 [2.6, 4.6]; 3 years: 3.0 [2.1, 4.3]; 5 years: 2.7 [1.5, 4.7]; all *p* < 0.001) (Table 2). Consistent results were observed when further stratifying patients with recurrence by recurrence type and separately estimating the associated HRs (Table S1). The Kendall's *τ* correlation between DFS and OS from initial nephrectomy was 0.70 (95% CI: 0.65, 0.74; *p* < 0.001), indicating a significantly positive association between DFS and OS.

**TABLE 2:** Cox proportional hazard model results of overall survival between patients without and with DFS after 1, 3, and 5 years following initial nephrectomy

Landmark point (years following initial nephrectomy)	Median OS after landmark point, years		Adjusted HR (95% CI)	*p*‐Value
	Without DFS (i.e., with recurrence)	With DFS (i.e., without recurrence)		
1 year	2.4	9.7	3.5 (2.6, 4.6)	<0.001
3 years	4.5	Not reached	3.0 (2.1, 4.3)	<0.001
5 years	5.7	Not reached	2.7 (1.5, 4.7)	<0.001

Abbreviations: CI, confidence interval; DFS, disease‐free survival; HR, hazard ratio; OS, overall survival.

### Multivariable regression analysis of incremental OS post‐nephrectomy associated with increased time to recurrence

**FIGURE 3:**
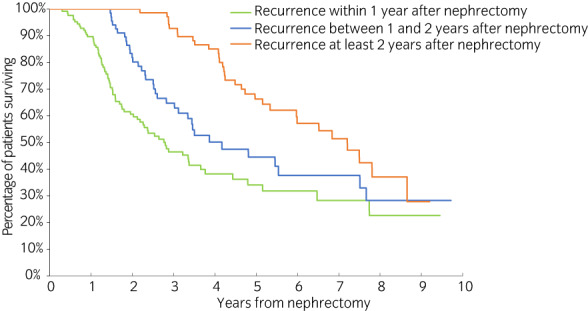
Overall survival stratified by time to recurrence among patients with recurrence.

Among patients with recurrence, those with longer time to recurrence were associated with longer OS (Figure 3). The adjusted analysis indicated that each additional year of DFS was associated with 0.73 additional years of OS post‐nephrectomy (95% CI: 0.40, 1.05 years; *p* < 0.001) (Table 3). Similar results were observed when more granular recurrence type was included as covariates (Table S2).

**TABLE 3:** Multivariable regression analysis of time to recurrence and overall survival (years)

	Coefficient (95% CI)	*p*‐Value
Unadjusted model	0.69 (0.40, 0.99)	<0.001
Adjusted model 1 (main analysis)^a^	0.73 (0.40, 1.05)	<0.001
Adjusted model 2 (sensitivity analysis)^b^	0.85 (0.52, 1.18)	<0.001

Abbreviations: CCI Charlson Comorbidity Index; CI, confidence interval; TNM, tumor‐node‐metastasis.

^a^ In the main analysis, the model adjusted for type of recurrence (i.e., metastasis vs. locoregional recurrence), year of recurrence, age at nephrectomy, type of nephrectomy (i.e., radical vs. partial), sex, race, CCI, disease stage at diagnosis (TNM staging), number of all‐cause inpatient visits per patient per month during the baseline period, and number of all‐cause outpatient visits per patient per month during the baseline period.

^b^ In the sensitivity analysis, the model adjusted for age at recurrence instead of age at nephrectomy. All other covariates adjusted for in the main analysis were also adjusted for.

When adjusting for age at recurrence instead of age at nephrectomy, the incremental OS increased from 0.73 to 0.85 years per one additional year of time to recurrence (95% CI: 0.52, 1.18 years; *p* < 0.001) (Table 3), reflecting a negative indirect effect from time to recurrence to post‐recurrence survival mediated through age.

## DISCUSSION

Regulators and payers often need to make market access, coverage, and reimbursement decisions using evidence based on intermediate endpoints, when OS data are immature. A well‐established association between intermediate endpoint and OS using IPD can help to better inform these decisions.^19^ To the best of our knowledge, the present study is the first to evaluate the association between DFS and OS among patients with intermediate‐high risk and high risk RCC post‐nephrectomy using real‐world IPD.

This study demonstrated that there was a significant positive relationship between DFS and OS among patients with RCC post‐nephrectomy, and that patients with recurrence post‐nephrectomy had roughly a three‐fold increased risk of death compared with patients without recurrence at the same timepoint. These findings indicate that, in this group of patients with intermediate‐high to high risk RCC post‐nephretomy, longer DFS is prognostic of longer OS, which helps to support use of DFS as a potential predictor of OS in patients with primarily resected RCC when OS data are immature.

A 2018 meta‐analysis from Harshman et al. used aggregated data from 13 published trials for adjuvant treatment for RCC to assess if DFS can be used as a surrogate for OS in the adjuvant setting.^14^ In that study, the R‐squared from the linear regression of treatment effects measured by DFS and OS was 0.44 (95% CI: 0.00, 0.69), which did not meet the priori chosen 0.7 threshold for a strong correlation.^14^ However, there is no universally established threshold for the strength of correlation between an intermediate endpoint and OS to establish surrogacy. It is important to note that Harshman et al. differs the present study in study design. For example, that meta‐analysis was based on aggregated data from published clinical studies with enrollment period ranging from late 1980s to late 2000s to evaluate the potential surrogacy of DFS for OS in localized RCC, whereas the present study used more recent, real‐world IPD to examine whether DFS was associated with OS among patients with intermediate‐high and high risk RCC post‐nephrectomy. Moreover, in the meta‐analysis most of the clinical trials reported null results for both DFS and OS, and none of the interventions (e.g., girentuximab, sorafenib) had significantly improved both DFS and OS relative to the comparators. As such, their findings may not be applicable to the scenario where novel treatments with potentially greater benefits in the adjuvant setting (e.g., immunotherapy) become available. Lastly, because survival post recurrence depends on real‐world availability of treatments for recurrent disease, their findings may not reflect the correlation between DFS and OS under the current treatment paradigm.

The results of the present study align with a study by George et al., which evaluated IPD from S‐TRAC.^25^ Results from that study demonstrated a correlation between DFS and OS. The estimated Kendall's *τ* between DFS and OS ranged from 0.51 to 0.88.^25^ Although evidence regarding the correlation of DFS and OS has not firmly established, the present study adds to the growing body of literature supporting the potential use of DFS as a predictor of OS when OS data are immature.

This study is associated with several limitations. First, the study only evaluated the correlation between DFS (or time to recurrence) and OS but did not establish any causal relationship. To obtain a comprehensive assessment of surrogate endpoints to inform future oncology studies, the correlation between treatment effects on DFS and OS would also need to be evaluated, which was not feasible in the current study due to the data limitation (only 16 patients receiving adjuvment treatments were identified from the data). Second, the administrative claims database used in this study did not have codes to directly identify RCC recurrence; therefore, the identification of recurrence relied on assumptions based on diagnosis codes, procedure codes, and drug codes. Thus, coding inaccuracies within the database may have led to misclassification bias and result in the erroneous identification of patients with recurrence. Third, the linked SEER‐Medicare database only includes Medicare patients who were ≥65 years; therefore, the findings from this study may not be generalizeable to younger patients with RCC or elderly patients not enrolled in Medicare. Future studies based on alternative real‐world data sources that include a younger patient population are warranted. Lastly, while these analyses were adjusted using key patient characteristics when feasible, the findings may be confounded by unmeasured characteristics, such as pathological features of the tumor and access to care, and thus causal inference should be drawn with caution.

The present study highlights the potential use of DFS as a predictor of OS when OS data are immature; however, the years of data analyzed in this study (2007–2016) do not capture the full treatment landscape for RCC to date. Although a few clinical trials investigating adjuvant treatments among patients with RCC have been conducted since 2004, it was not until 2017 that the FDA approved the first adjuvant treatment for RCC.^26^ Therefore, the results from the current study do not reflect the association between DFS and OS post‐nephrectomy in the era of adjuvant therapy. Future studies using more up‐to‐date real‐world data could complement the current findings, as real‐world evidence on the use of adjuvant therapy for RCC accumulate. Likewise, during the study period, novel treatments for metastaic RCC (e.g., immuno‐oncology therapies), had not become the standard treatment for patients with advanced disease. Thus, the findings from the current study may not be applied to patients receiving such novel treatments these days.

## AUTHOR CONTRIBUTIONS

**Naomi B. Haas:** Conceptualization; formal analysis; investigation; methodology; resources; supervision; validation; writing – original draft; writing – review and editing. **Yan Song:** Conceptualization; data curation; formal analysis; funding acquisition; investigation; methodology; project administration; resources; supervision; validation; visualization; writing – original draft; writing – review and editing. **Jaqueline Willemann Rogerio:** Conceptualization; formal analysis; funding acquisition; investigation; methodology; resources; supervision; validation; writing – original draft; writing – review and editing. **Su Zhang:** Conceptualization; data curation; formal analysis; funding acquisition; investigation; methodology; project administration; resources; software; supervision; validation; visualization; writing – original draft; writing – review and editing. **Christopher Carley:** Conceptualization; data curation; formal analysis; investigation; methodology; project administration; resources; software; validation; visualization; writing – original draft; writing – review and editing. **JingJing Zhu:** Conceptualization; data curation; formal analysis; investigation; methodology; project administration; resources; software; validation; visualization; writing – original draft; writing – review and editing. **Rituparna Bhattacharya:** Conceptualization; formal analysis; funding acquisition; investigation; methodology; project administration; resources; supervision; validation; visualization; writing – original draft; writing – review and editing. **James Signorovitch:** Conceptualization; data curation; formal analysis; funding acquisition; investigation; methodology; project administration; resources; supervision; validation; writing – original draft; writing – review and editing. **Murali Sundaram:** Conceptualization; formal analysis; funding acquisition; investigation; methodology; project administration; resources; supervision; validation; visualization; writing – original draft; writing – review and editing.

## CONFLICT OF INTEREST

NBH is a paid consultant to Roche Genentech, Exilexis, Aveo, and Merck & Co., Inc. YS, SZ, CC, and JS are paid employees of Analysis Group, Inc. which received consultancy fees from the study sponsor for the conduct of this study. JZ was a paid employee of Analysis Group, Inc. at the time this study was conducted. JWR, RB, MS are employees of Merck Sharp & Dohme LLC, a subsidiary of Merck & Co., Inc., Rahway, NJ, USA and may own stock and/or stock options in Merck & Co., Inc., Rahway, NJ, USA. This study was funded by Merck Sharp & Dohme LLC, a subsidiary of Merck & Co., Inc., Rahway, NJ, USA.

## PREVIOUS PRESENTATIONS

Portions of this research were presented in poster form at the American Society of Clinical Oncology (ASCO) 2021 Annual meeting on June 4–8, 2021 (virtual congress).

## APPROVAL OF THE RESEARCH PROTOCOL BY AN INSTITUTIONAL REVIEWER BOARD

The New England Independent Review Board exempted this study from institutional board review on January 13, 2020.

## INFORMED CONSENT

Not applicable.

## REGISTRY AND THE REGISTRATION NO. OF THE STUDY/TRIAL

Not applicable.

## ANIMAL STUDIES

Not applicable.

## Supporting information


Appendix S1.

## Data Availability

The data that support the findings of this study are available from the National Cancer Institute. Restrictions apply to the availability of these data, which were used under license for this study.
