# Supplementary material for: Disease‐free survival as a predictor of overall survival in localized renal cell carcinoma following initial nephrectomy: A retrospective analysis of Surveillance, Epidemiology and End Results‐Medicare datac
Source: Int J Urol. 2023 Feb 14;30(3):272–9. doi: 10.1111/iju.15104 (PMC11524080; doi:10.1111/iju.15104)
Supplement: Supplementary file 1 — Appendix S1. [file IJU-30-272-s001.docx]

# **Supplemental Materials**

## **Supplemental Table 1. Cox Proportional Hazard Model Results of Overall Survival between Patients without and with DFS after 1, 3, and 5 Years following Initial Nephrectomy**

| **Landmark point (years following initial nephrectomy)** | **DFS Status** | **Adjusted HR**  **(95% CI)** | **P-value** |
| --- | --- | --- | --- |
|  |  |  |  |
| 1 year | Locoregional recurrence | 1.68 (0.82, 3.45) | 0.157 |
|  | Metastasis with single metastatic site - lung | 4.84 (2.99, 7.81) | <0.001 |
|  | Metastasis with single metastatic site - other | 3.17 (2.14, 4.71) | <0.001 |
|  | Multiple metastatic sites | 16.26 (7.40, 35.74) | <0.001 |
| 3 years | Locoregional recurrence | 1.33 (0.57, 3.07) | 0.508 |
|  | Metastasis with single metastatic site - lung | 5.59 (3.31, 9.44) | <0.001 |
|  | Metastasis with single metastatic site - other | 2.44 (1.51, 3.95) | <0.001 |
|  | Multiple metastatic sites | 29.62 (10.98, 79.88) | <0.001 |
| 5 years | Locoregional recurrence | 2.13 (0.81, 5.61) | 0.125 |
|  | Metastasis with single metastatic site - lung | 3.14 (1.09, 9.06) | 0.034 |
|  | Metastasis with single metastatic site - other | 2.84 (1.49, 5.39) | 0.001 |
|  | Multiple metastatic sites^†^ | - | - |

CI, confidence interval; DFS, disease-free survival; HR, hazard ratio; OS, overall survival.

^†^HR associated with patients with multiple metastatic sites is not estimated due to the small sample size of patients after 5 years following initial nephrectomy.

## **Supplemental Table 2. Multivariable Regression Analysis of Time to Recurrence and Overall Survival (years)**

|  |  | **Coefficient (95% CI)** | **P-value** |
| --- | --- | --- | --- |
|  |  |  |  |
|  | Adjusted model 3 (sensitivity analysis) | 0.65 (0.34, 0.97) | <0.001 |
| CI, confidence interval; | | | |

^†^In this sensitivity analysis, the model adjusted for type of recurrence (i.e., locoregional recurrence vs. metastasis with single metastatic site at lung vs. metastasis with single metastatic site at other organs vs. metastasis with multiple metastatic sites), year of recurrence, age at nephrectomy, type of nephrectomy (i.e., radical vs. partial), sex, race, CCI, disease stage at diagnosis (TNM staging), number of all-cause inpatient visits per patient per month during the baseline period, and number of all-cause outpatient visits per patient per month during the baseline period.
